# Transposable element variation in Arabidopsis: Far Eastern accessions are loaded up

**DOI:** 10.1093/plcell/koad306

**Published:** 2023-12-11

**Authors:** Johan Zicola

**Affiliations:** Assistant Features Editor, The Plant Cell, American Society of Plant Biologists; Department of Crop Sciences & Center for Integrated Breeding Research, The University of Göttingen, 37075 Göttingen, Germany

Evolution is driven by changes in DNA sequence, including single nucleotide mutations and larger sequence changes such as structural variants. Transposable elements (TEs) are mobile DNA sequences that can insert in coding and regulatory regions, potentially altering gene function. Due to their disruptive effect on genome integrity, most TEs are kept silent. However, transposition events still occur and may increase the genetic load.

In this study, **Juan Jiang and coauthors (**Jiang et al. 2023**)** assessed TE variation in a worldwide collection of 1,114 genome-sequenced Arabidopsis accessions, including 870 accessions from the 1001 Genome Project and Africa and 244 Chinese accessions sequenced by the authors. Since nonrelict Arabidopsis expanded along the East-West axis from the Balkans, these additional Chinese accessions provide a better view on the Far Eastern expansion front of the species.

The authors first described the variation in polymorphic TE numbers across all accessions using the European accession Col-0 as reference. Using paired-end short-read sequencing data, they identified a total of about 43,000 polymorphic TEs with a range of ≍5,000 to 6,500 polymorphic TEs per accession. The repetitive nature of TE sequences often leads to false-positive results due to sequencing read mapping ambiguities and reference biases. Therefore, the authors used long-read–based genome assemblies of 9 accessions belonging to different populations and found that an average of 66% presence-absence variations called with short-read sequencing data are true positive, with variation across TE families and genomic regions. They also found that polymorphic TE number is robust when using different genome assemblies as references.

To assess the transposition activity of polymorphic TEs, the authors looked at TE expression in DNA methylation mutants (Col-0 background) where TE silencing is partially released. Interestingly, they found an enrichment of polymorphic TEs expressed in the TE-activated mutants, supporting their potential active state in the natural accessions examined.

To assess the deleterious effect of TEs at the genomic level, the authors compared the age-adjusted site frequency spectrum between TEs and other coding single nucleotide polymorphisms. They found that intragenic TEs and TEs located near functionally important genes tend to be more deleterious. They also found that synergistic epistasis is present among TEs with more deleterious effects.

Looking at the TE load in different populations, the authors found that TEs accumulate along the range expansion axis and reach the highest abundance in accessions that are at the Far Eastern front expansion (Chinese accessions) (Fig. 1). Effective population size (*N_e_*) could explain 62.0% variation of the TE load (Fig. 1). The authors suggested that high transposition rate and selective sweeps also contributed to the high TE load within the Yangtze River basin population, implying a potentially adaptive function associated with TE accumulation in this population.

**Figure 1.**
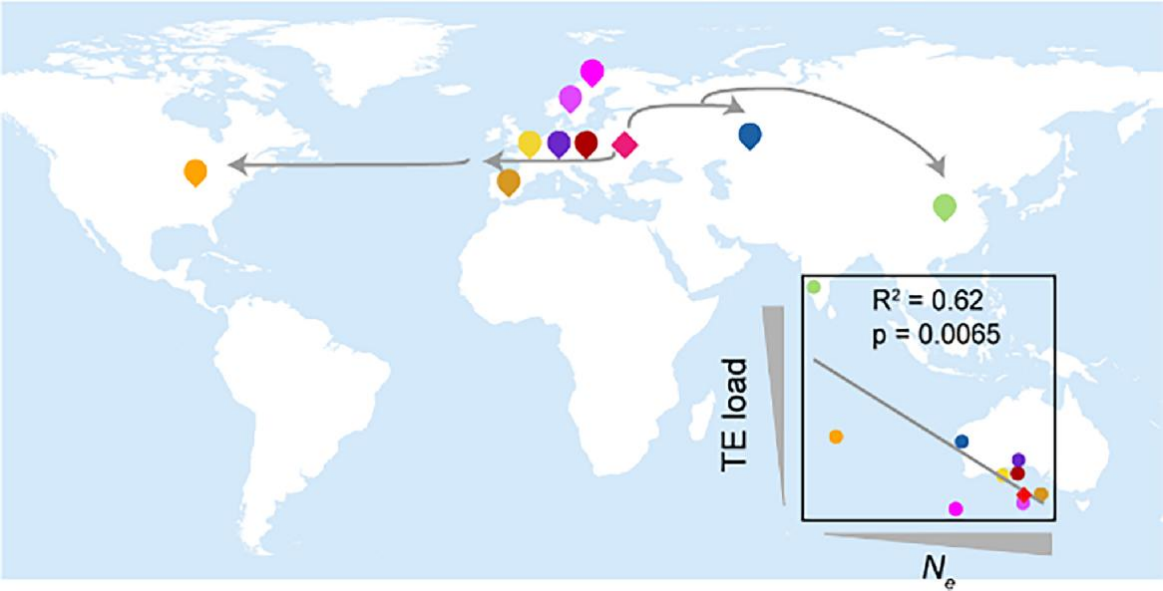
TE load increased while effective population size (*N*_*e*_) decreased during Arabidopsis range expansion. Adapted from Jiang et al. (2023), Figure 3.

To reveal the genetic architecture involved in TE load variation, the authors performed genome-wide association studies (GWAS) for each of the 319 TE families using as phenotypes expression levels and copy number variation. The GWAS for TE family expression and copy number resulted in 209 and 315 peaks, containing 3,640 and 651 candidate genes and 81 and 9 TEs, respectively. No specific gene ontology terms were enriched within the candidate genes. Intriguingly, 139 candidate genes identified in the GWAS for both phenotypes were enriched in the salicylic acid signaling pathway, indicating that this pathway could have a role in TE regulation. In addition, 1 strong candidate gene was found in the GWAS for ATHILA4D TE family expression: *RNA-dependent RNA polymerase 2* (*RDR2*), known to be involved in the epigenetic silencing of TEs. Also, the missense mutation found at *RDR2* was present at a high frequency in the population of the Yangtze River basin, where relative TE load is the highest. Overall, the genetic architecture of TE variation in Arabidopsis seems to be highly polygenic.

Building on previous research (Quadrana et al. 2016; Stuart et al. 2016; Baduel et al. 2021) with the addition of newly sequenced Chinese accessions, the authors described the genetic architecture and evolutionary drivers of TE load variation during Arabidopsis range expansion. Further research will hopefully define the molecular mechanisms involved in the high-TE load in Far Eastern accessions and its evolutionary consequences.

## Dive Curated Terms

The following phenotypic, genotypic, and functional terms are of significance to the work described in this paper:

RDR2 Gramene: AT4G11130
RDR2 Araport: AT4G11130
